# Organochlorine pesticides (OCPs) in wetland soils under different land uses along a 100-year chronosequence of reclamation in a Chinese estuary

**DOI:** 10.1038/srep17624

**Published:** 2015-12-03

**Authors:** Junhong Bai, Qiongqiong Lu, Qingqing Zhao, Junjing Wang, Zhaoqin Gao, Guangliang Zhang

**Affiliations:** 1 State Key Laboratory of Water Environment Simulation, School of Environment, Beijing Normal University, Beijing 100875, P.R. China

## Abstract

Soil profiles were collected at a depth of 30 cm in ditch wetlands (DWs), riverine wetlands (RiWs) and reclaimed wetlands (ReWs) along a 100-year chronosequence of reclamation in the Pearl River Delta. In total, 16 OCPs were measured to investigate the effects of wetland reclamation and reclamation history on OCP levels. Our results showed that average ∑DDTs, HCB, MXC, and ∑OCPs were higher in surface soils of DWs compared to RiWs and ReWs. Both D30 and D20 soils contained the highest ∑OCP levels, followed by D40 and D100 soils; lower ∑OCP levels occurred in D10 soils. Higher ∑OCP levels were observed in the younger RiWs than in the older ones, and surface soils exhibited higher ∑OCP concentrations in the older ReWs compared with younger ReWs. The predominant percentages of γ-HCH in ∑HCHs (>42%) and aldrin in ∑DRINs (>46%) in most samples reflected the recent use of lindane and aldrin. The presence of dominant DDT isomers (*p,p’*-DDE and *p,p’*-DDD) indicated the historical input of DDT and significant aerobic degradation of the compound. Generally, DW soils had a higher ecotoxicological risk of OCPs than RiW and ReW soils, and the top 30 cm soils had higher ecotoxicological risks of HCHs than of DDTs.

Organochlorine pesticides (OCPs) have caused increasing global concern due to their high toxicity, persistence, bioaccumulation and significant adverse effects on ecosystems and human health through food chains^1^,^2^,^3^,^4^. The wide application of pesticides across the globe, especially in developing countries to maximize crop yields, has resulted in extensive contamination worldwide^5^,^6^. OCPs have a strong affinity for suspended particulate matter and are subsequently deposited into river and marine sediments or wetland soils due to their low water solubility and high hydrophobicity^4^,^7^. Consequently, the residues of OCPs in soil/sediment can serve as a useful indicator for aquatic ecosystem health^2^ because low OCP levels might cause adverse biological effects^8^. Therefore, an investigation into the occurrence, sources and ecological risks of OCPs in wetland soils can contribute to a better understating of the status of OCP pollution in wetlands and can aid in maintaining wetland ecosystem health and ecological safety.

Land use changes, especially the conversion of natural ecosystems to croplands, can greatly influence the levels and distribution of OCPs because soil tillage can enhance the exchange at the interface of soil and air through changing soil properties^9^. Residues of OCPs have been widely reported to be at higher levels in agricultural soils than in forest soils^10^, soils from unused land or fallow land^11^,^12^,^13^. Wang *et al*.^5^ observed lower OCP levels in wetland soils compared with other land use types. Additionally, changing land from a rice paddy to a vegetable field increased the residues of OCPs in the first 15 years due to changes in anaerobic and aerobic conditions but ultimately exhibited a decrease in residue levels from 20 to 30 years^14^. The above studies have focused on the investigation of OCP levels in agricultural soils^11^,^12^,^14^, forest soils^9^,^10^ and wetland soils^5^; however, little information is available on the changes in OCP levels after the conversion of wetlands to croplands due to the effects of tillage history.

OCPs have been widely applied across China in the past decades, and the total production of HCHs and DDTs in China was approximately 4.9 and 0.4 million tons, respectively, before their official ban in 1983^15^. Additionally, approximately 3,200 tons of lindane (nearly pure HCH) was still in use in China from 1991 to 2000^16^. Dicofol with a high level of impurity of DDT compounds, is still allowed and widely used in agricultural practice like cotton cultivation in China after the official ban in 1983^17^. Some studies have reported that the largest amount of pesticide application occurred in Southeast China in places like the Pearl River Delta^14^,^18^. High residues of OCPs (nd-649.33 ng g^−1^) have been reported in agricultural soils in the Pearl River Delta^11^,^19^, of which DDTs (average 68.5 ng g^−1^) and HCHs (average 16.2 ng g^−1^) were the dominant pollutants, and paddy soils were shown to be heavily DDT-contaminated soils (>40 ng g^−1^)^15^. Additionally, DDT levels in sediments of the Zhujiang rivers (35.1–91.0 ng g^−1^) fell among the highest values of the worldwide concentration range^15^ due to the considerable load of chlorinated pesticides (up to 863 tons per year) in the Pearl River^20^. The Pearl River Delta has been well developed agriculturally and many of its coastal wetlands have been reclaimed over the last 100 years^21^, which has consumed the highest amount of pesticides per unit of agricultural land (37.2 kg ha^−1^) in the country^22^. This occurrence might contribute substantially to global OCP cycles^23^ because OCPs can be dispersed and redistributed from lower to higher altitudes on a global scale^24^. However, the effects of wetland reclamation and of reclamation history on the OCP levels found in soils subjected to different land uses remain unknown.

The primary objectives of this study were (1) to investigate the occurrence and profile distributions of OCPs in soils under different land uses (i.e., ditch wetlands, riverine wetlands, and reclaimed wetlands) along a 100-yr chronosequence of reclamation in the Pearl River Estuary; (2) to identify the potential sources of OCPs in soils under different land uses; and (3) to assess the ecotoxicological risks of OCPs in soils with different reclamation histories.

## Results and Discussion

### Occurrence of OCPs in soil profiles under different land uses

**Figure 1:**
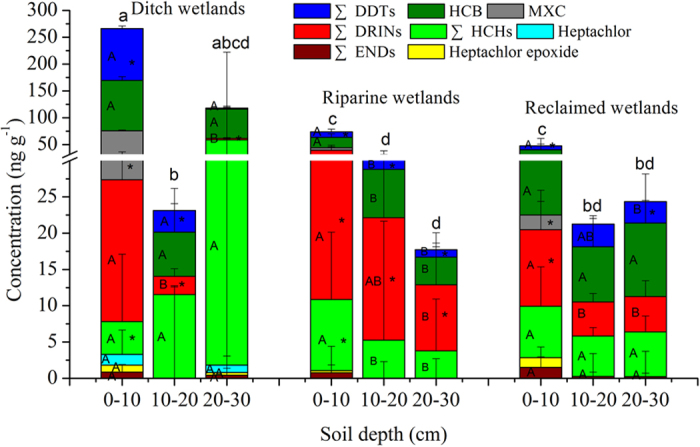
The average levels of OCPs in soil profiles under different land uses. ^*^The bars with the same colour in the same soil depth represent significant differences in OCP isomers among different wetland types (*p* < 0.05). ^AB^Different letters in the bars with the same colour in the same wetland type represent significant differences in OCP isomers among different soil depths (*p* < 0.05). ^abcd^Different letters on the top of bars represent significant differences in ∑OCP levels among different wetlands (*p* < 0.05).

Figure 1 shows the average levels of OCP isomers and ∑OCPs in the soil profiles of different wetlands. Generally, the surface soils (0–10 cm) in DWs contained higher ∑OCP and MXC levels than those from RiWs and ReWs, and lower ∑DRIN levels (in particular aldrin, Supplementary information Table S1) were observed in ReWs than in RiWs (*p *< 0.05, Fig. 1). Higher SOC contents in DWs than in RiWs and ReWs^25^ could greatly contribute to OCP accumulation due to their high affinity for organic matter^26^. RiW surface soils contain higher levels of ∑HCHs (especially γ-HCH, Supplementary information Table S1) than do DW surface soils (*p* < 0.05). DWs were found to have higher ∑DDT levels and lower ∑DRIN and ∑OCP levels in the 10–20 cm soil layer and lower ∑DRIN levels in the 20–30 cm soil layer than RiWs (*p* < 0.05). However, ∑DDT levels were higher in the 20–30 cm soil layer in ReWs compared with RiWs (*p* < 0.05). This implies that more OCPs had accumulated in ditch wetland soils affected by the agricultural and sewage drainage from residents living on both banks of the ditches^21^. The average total OCPs found in the surface soils (0–10 cm) were 266.18 ng g^−1^ in ditch wetlands, 73.67 ng g^−1^ in RiWs and 47.69 ng g^−1^ in ReWs (Supplementary information Table S1). These levels are much higher than those found in the wetland soils of the Yellow River Delta (1.68 ng g^−1^)^27^. Compared with the Yangtze River Delta (88.8 ng g^−1^)^28^, the average level of ∑DDTs in DW soils was higher, whereas they were lower in RiW and ReW soils.

As shown in Fig. 1, surface soils (0–10 cm) contained much higher levels of ∑DRINs and MXC than deeper soils in DWs (*p* < 0.05). We also observed a decrease in the average levels of ∑DDTs, HCB, ∑ENDs, and heptachlor epoxide and an increase in ∑HCHs along the soil profiles in DWs despite of no significant differences (*p* > 0.05). All OCP isomers including ∑DDTs, HCB, MXC, ∑ENDs, ∑DRINs,∑HCHs, heptachlor epoxide, and heptachlor exhibited higher average levels in surface soils than those found in deeper RiW soils (*p* < 0.05). In ReWs, surface soils also contained higher levels of ∑DDTs, MXC, ∑DRINs, and heptachlor epoxide compared with deeper soils (*p* < 0.05). Generally, upper soils contained much higher average levels of ∑OCPs than deeper soils in the three wetlands (*p* < 0.05; Fig. 1). This might be associated with new sources of OCPs that were introduced after 1983^26^ or by their continuous usage for other purposes in addition to their agricultural purposes^29^. The usage of HCB and some DDTs was not completely terminated until 2009, and endosulfan and dicofol might be still produced and used in China^11^,^17^. Additionally, technical DDT was still used for the production of antifouling paints under an exemption of the Stockholm Convention^30^.

### Profile distribution of OCPs in three wetlands with different reclamation histories

**Figure 2:**
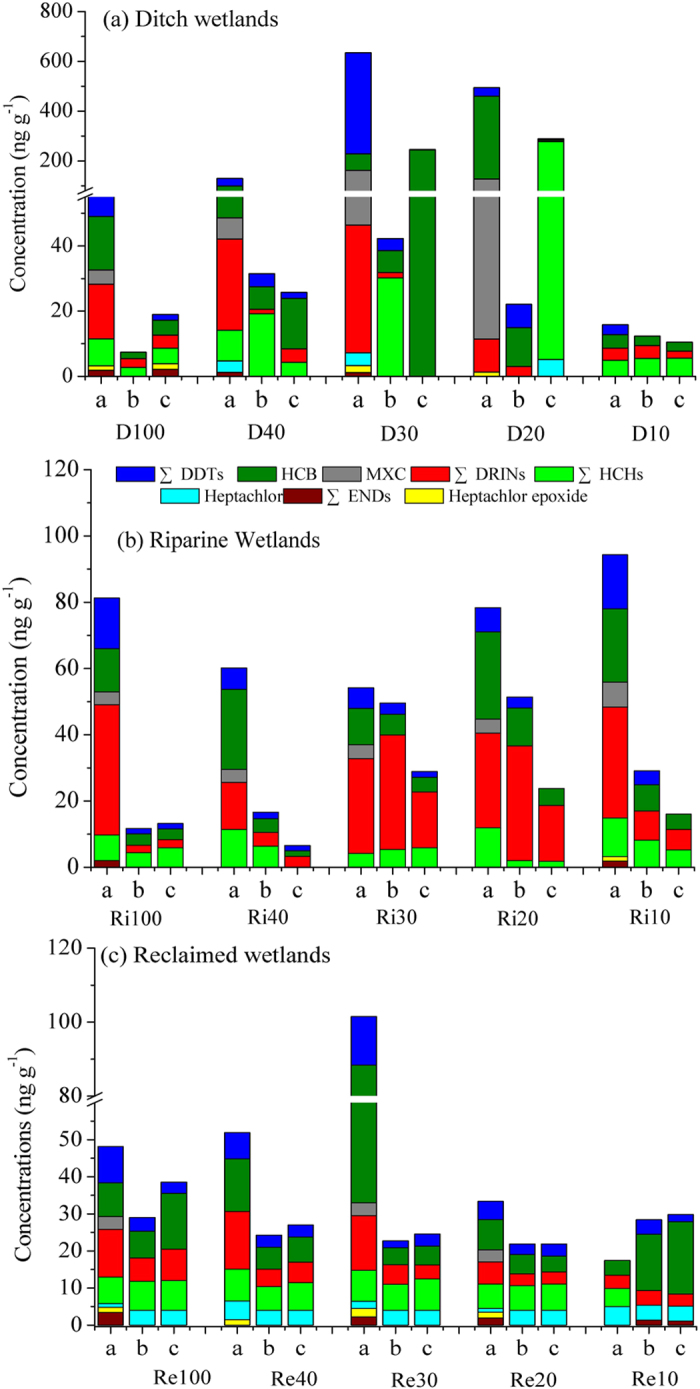
Profile distribution of OCPs in soils under different land uses along a 100-yr chronosequence of reclamation. (**a–c**) present 0–10 cm, 10–20 cm, and 20–30 cm, respectively.

The profile distribution of OCPs in ditch wetlands, riverine wetlands and reclaimed wetlands are plotted in Fig. 2. As shown in the Figure, ∑DDTs, ∑DRINs, MXC, and ∑OCPs generally decreased with depth along soil profiles at most sampling sites in these three wetlands. The dominant OCP isomers included ∑HCHs, ∑DDTs, HCB, and ∑DRINs in each of the three wetlands. The lower concentrations of the rest OCP isomers might be associated with their shorter production history and lower popularity in their usage^26^.

Among the five DW sampling sites, the highest concentrations of ∑DDTs (407.44 ng g^−1^) and HCB (65.18 ng g^−1^) were observed in the D30 and D20 surface soils, respectively. This was associated with the fact that DDTs and HCB were not completely terminated until May of 2009 in China^11^. Meanwhile, HCB has also been detected as an impurity in some pesticides, such as lindane, due to its formation as a by-product during the production process^31^. Both the MXC and ∑ENDs concentrations were also higher in the D30 and D20 surface soils. This implies that more MXC and endosulfan might have been used and accumulated in these regions because MXC is an insecticide that was developed to replace DDT and was widely used all over the world because of its lower toxicity to mammals and lower persistence in the environment relative to DDT^26^,^32^. Meanwhile, endosulfan is still in use in China^12^. Additionally, we also observed the highest ∑HCHs concentrations in the 20–30 cm soil layer of the D20 wetlands. Therefore, both D30 and D20 soils contained high levels of ∑OCPs, followed by D40 and D100 soils, whereas the D10 soils contained the lowest amounts. This indicates higher OCP accumulation in the ditch wetlands with >20 reclaimed histories due to higher input before the 2000s^26^. Higher HCB (in D30) and ∑HCHs (in D20) in the 20–30 cm soil layer reflects more extensive use of OCPs in China in the past. Additionally, these contaminants could enter the aquatic ecosystems through effluent discharge, atmospheric deposition, runoff, and other pathways^7^.

In RiWs, ∑DRINs accounted for a greater percentage of the OCP composition in almost all soils. The ∑OCPs in surface soils decreased before increasing from Ri100 to Ri10 wetlands, with lower ∑OCPs in Ri30. However, higher ∑OCPs occurred in deeper soils of younger riverine wetlands (i.e., Ri30, Ri20 and Ri10) compared to older riverine wetlands (i.e., Ri100 and Ri40). Similarly, in ReWs, ∑DRINs, ∑HCHs, and HCB were the dominant OCP isomers, and total OCP concentrations were generally higher in surface soils than those in deeper soils, except for higher HCB concentrations found in deeper soils in the Re10 wetlands. Higher ∑OCP concentrations were present in the surface soils of older reclaimed wetlands (from Re30 to Re100) compared to the younger reclaimed wetlands (i.e., Re20 and Re10). This might be associated with higher production and wider usage of these in agricultural soils before the 1980s in China^26^,^33^, and the official ban of HCHs and DDTs in 1983^15^. This indicates higher accumulation of ∑OCPs, especially HCB and∑DRINs, in older reclaimed wetlands.

We observed higher ∑OCP levels in the surface soils of DWs in the 20-yr, 30-yr and 40-yr reclaimed regions (i.e., D40, D30, and D20) compared with RiWs and ReWs. This could be attributed to different land-use changes because land use has a direct impact on the application history and the dissipation of OCPs by changing the soil conditions^33^,^34^. However, in the 10-yr and 100-yr reclaimed regions, ∑OCP levels in surface soils were lower in the DW (i.e., D10 and D100) and ReW soils (i.e., Re10 and Re100) than in RiW soils (i.e., Ri10 and Ri100). This finding implies less OCP application in the Re10 and Re100 wetlands and lower accumulation in the D10 and D100 wetlands; however, higher OCP levels in the Ri10 and Ri100 wetlands might come from upstream inputs due to a considerable load of chlorinated pesticides (up to 863 tons per year) in the Pearl River^20^.

### Composition and potential sources

**Figure 3:**
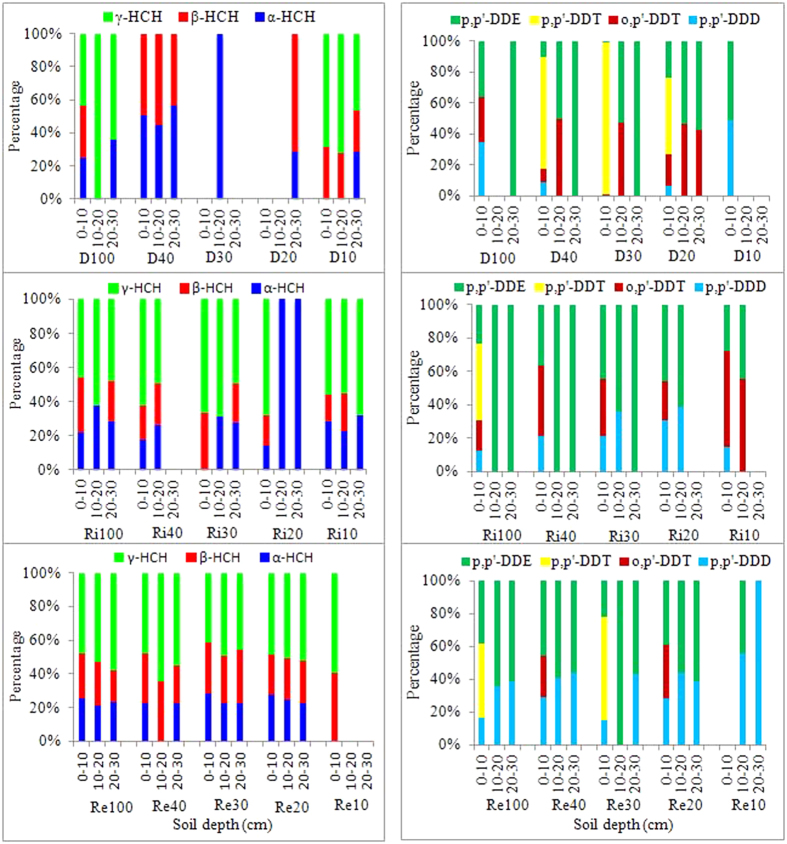
Profile distribution of the percentages of the composition of HCH or DDT isomers in soils under different land uses along a 100-yr chronosequence of reclamation.

The composition percentages of HCH or DDT isomers in soils can be used to indicate different sources of contamination^2^,^16^. Typical technical HCH formulations contain 60–70% *α*-HCH, 5–12% *β*-HCH, 10–15% *γ*-HCH, and 6–10% *δ*-HCH^2^. As shown in Fig. 3, β-HCH was present in high ratios at most sampling sites in ditch wetlands (i.e., D40, D30, and D20) and reclaimed wetlands. This finding suggests that there have been no fresh inputs of technical HCHs because *β*-HCH is the most stable isomer and relatively resistant to microbial degradation due to its low water solubility and vapour pressure^35^. Moreover, both *α*-HCH and *γ*-HCH can be converted to *β*-HCH once in the environment^36^. However, the predominant percentage of *γ*-HCH ranging from 42% to 100% in most soil samples from the DWs (i.e., D100 and D10), RiWs and ReWs reflects the more common use of lindane rather than technical HCH in the region because *γ*-HCH accounts for more than 99% of lindane^2^,^26^. Meanwhile, the ratios of *α*-HCH to *γ*-HCH (varying from 0 to 0.68) in these soil samples were considerably low compared with the ratios of *α*-HCH to *γ*-HCH in technical HCHs (from 3 to 7)^37^, which also implies the use of lindane, because low *α*-HCH/*γ*-HCH ratios (close to 0) reflect the recent input of lindane^11^,^26^.

Technical DDTs generally contains 75% *p,p’*-DDT, 15% *o,p’*-DDT, 5% *p,p’*-DDE and <5% *p,p’*-DDD^38^. Both *p,p’*-DDE and *p,p’*-DDD were the dominant DDT isomers in three soil layers of most of the sampling sites in the DWs, RiWs and ReWs, with exception of the surface soils (0–10 cm) of the D40, D30, D20, Ri100 and Re30 wetlands (Fig. 3). This indicates that the input of DDTs in the land was historical, as DDTs can be biodegraded to DDE under aerobic conditions and to DDD under anaerobic conditions^38^. A ratio of (DDE+DDD)/∑DDT>0.5 reflects long-term weathering^39^,^40^. With the exception of the abovementioned surface soil samples, a ratio of (DDE+DDD)/∑DDT>0.5 was obtained at most sampling sites, suggesting that the input of DDTs to these wetlands, especially the RiWs and ReWs, was historical and significant degradation has occurred since the official ban of DDTs in 1983^2^. However, fresh inputs might be occurring to the surface soils from D40, D30, D20, Ri100 and Re30. Zhou *et al*.^41^ reported that the illegal application of DDT as part of agricultural activities still occurred in some regions of the Pearl River Delta until the end of 2000^42^. Additionally, Liu *et al*.^17^ reported that dicofol, with high levels of DDT impurities, is still allowed and widely used in agricultural practices like cotton cultivation in China. Compared with *p,p’*-DDD, higher ratios of *p,p’*-DDE (ranging from 43% to 100%) occurred in subsurface soils, except for in the 20–30 cm soil layer of the Re10 wetlands, indicating the dominance of aerobic DDT biodegradation^38^.

Levels of *o,p*-DDT exceeding those of *p,p*-DDT were measured in some surface soil samples, especially in RiWs. This is very different from the composition of technical DDT and might be related to the dicofol (containing 11.4% *o,p*-DDT and 1.7% *p,p*-DDT) applied to agricultural areas since the official ban of DDTs in 1983^43^. The DDE/DDD ratio is an indicator of the aerobic/anaerobic conditions present in the sediment environment^17^,^44^. With the exception of the 20–30 cm soil layer in the Re10 wetlands, the DDE/DDD ratios in almost all of the soil samples were above 1, which further indicates that relatively aerobic conditions exist in these sedimentary environments^26^,^44^.

**Figure 4:**
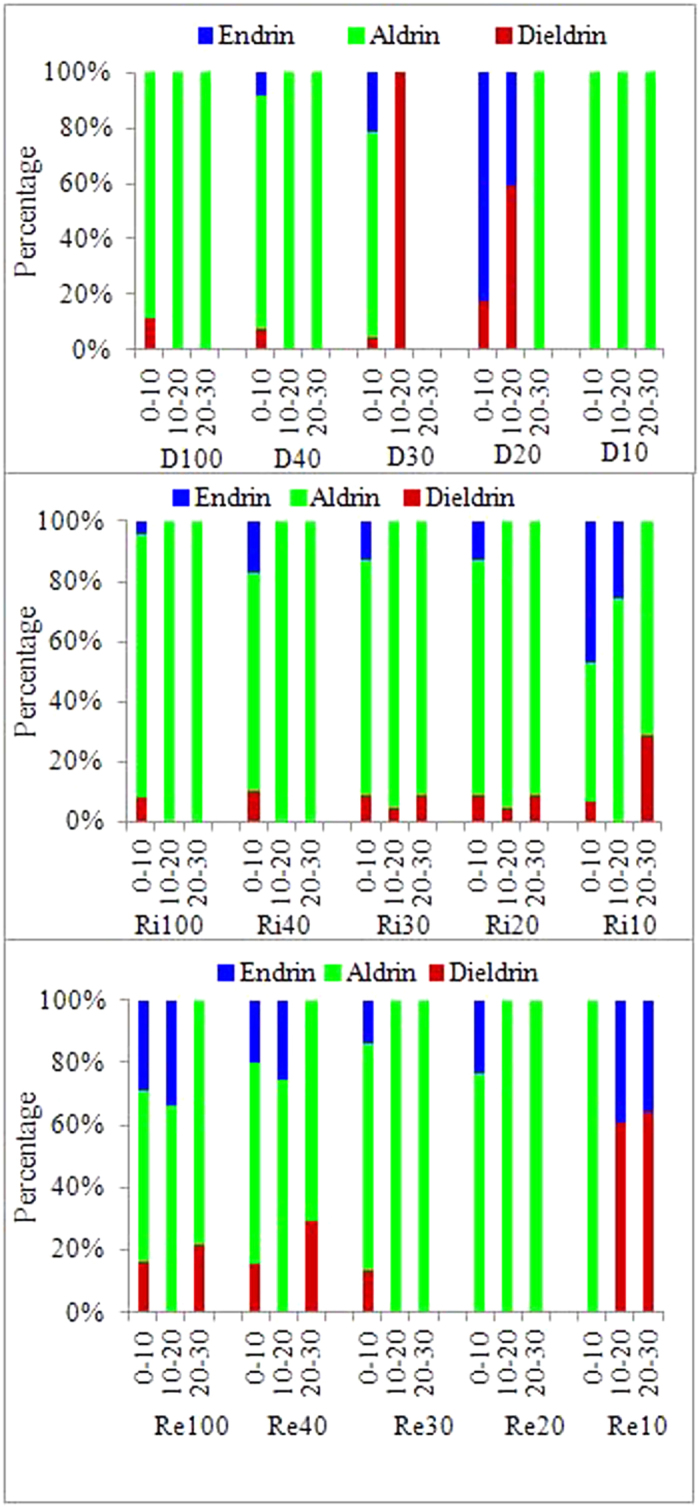
Profile distribution of the percentages of the composition of DRIN isomers in soils under different land uses along a 100-yr chronosequence of reclamation.

Aldrin was the dominant isomer of the ∑DRINs in all soil samples except for the 10–20 cm soil layers of the D30 wetlands and the 0–20 cm soil layers of the D20 and Re10 wetlands (Fig. 4). Aldrin can be quickly converted to dieldrin by sunlight and bacteria in the environment, and dieldrin can subsequently remain in the soil for a long time by adhering to the soil^45^,^46^. However, in these three wetlands, the percent composition of aldrin ranged from 46% to 100% for most soils, reflecting a fresh source of aldrin input in this region^46^. In contrast, the higher percent composition of dieldrin in the 10–20 cm soil layers of D30 (100%) and D20 (59%) and in the 10–30 cm soil layers of Re10 (>60%), indicating the historical conversion of aldrin to dieldrin. In the D20 wetlands, endrin was accumulated in the surface soils at 82% of ∑DRINs, even though it has been officially banned from use since 1983^26^. This indicates that there is a new technical input of endrin in this region and that its potential sources in the region near the D20 wetlands need to be further investigated.

### Ecotoxicological Risk Assessment

**Figure 5:**
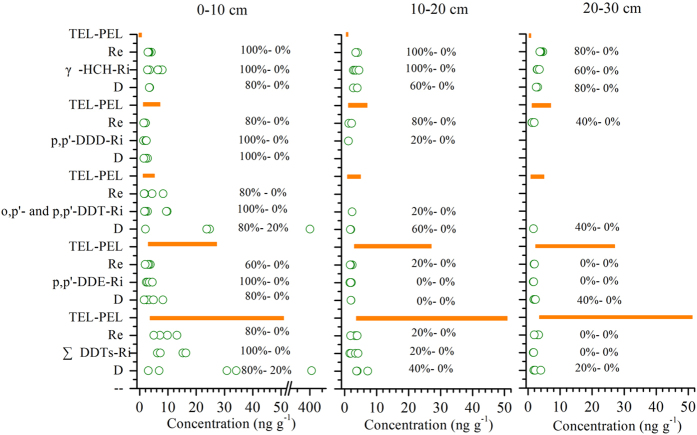
OCPs concentration in different soil layers from three wetlands as compared to the TEL and PEL. (Threshold effect levels and probable effect levels were suggested by Long *et al*.^37^). Grey bars denote TEL-PEL, and %–% represents the percentage in excess of TEL and PEL.

The data collected from the three wetlands were compared with data obtained from the US-EPA/NOAA^47^ for marine sediments (Fig. 5). The NOAA guidelines specify threshold effect levels (TELs) and probable effect levels (PELs). The TELs represent low threshold concentrations below which adverse effects on sediment-dwelling fauna would be rarely expected, whereas the PELs represent the concentrations above which adverse effects are probable^47^. According to the guidelines, all surface soils in the RiWs and more than 60% of the surface soils in ReWs were grouped into TEL-PEL subsets. Similarly, 80% of the surface soils in DWs exceeded the TEL levels, and even 20% of surface soils were above the PEL levels of ∑DDTs and the sum of *o,p’*-and *p,p’*-DDT, which would have an adverse effect on sediment-dwelling organisms. Moreover, one soil sample from the D30 wetlands exhibited the highest ∑DDTs level (96.48 ng g^−1^), which exceeded the limit of DDTs in birds (11 ng g^−1^) and soil biological communities (10 ng g^−1^)^48^. In the 10–20 cm soil layer, 40–60% of soil samples in DWs and 0–80% of soils in ReWs and RiWs were below the TEL levels^47^, indicating that they had no or a lower adverse effect on sediment-dwelling organisms in subsurface soils. In the 20–30 cm soil layer, the concentrations of *p,p’*-DDE, ∑DDTs and the sum of *o,p’*-and *p,p’*-DDT in the ReWs and RiWs were below the TELs in all soil samples. However, in the DWs, approximately 20–40% of soils in the 20–30 cm soil layers exceeded the TEL levels for DDTs^47^.

Approximately 60–100% of the soil samples in the three wetlands were above the TELs for *γ*-HCH, particularly in the upper soils of the ReWs and RiWs. These soils exhibited higher risks because the*γ*-HCH levels in all of their soil samples were grouped into TELs-PELs. However, all of the*γ*-HCH values were below the limit (80 ng g^−1^) of risk for 10% of species^49^. According to the Dutch target values^50^, the residues of HCHs in approximately 13–20% of soil samples in DWs and RiWs exceeded the target value for unpolluted soil (10 ng g^−1^)^50^, whereas all the ReW soils exhibited lower levels than the target value. For DDTs, more than 50% of soil samples in three wetlands exceeded the target value for unpolluted soil (2.5 ng g^−1^)^50^. Generally, in the three wetlands, the top 30 cm soils showed higher ecotoxicological risks of HCHs compared to DDTs. Ditch wetlands showed higher ecotoxicological risks of OCPs than riverine and reclaimed wetlands. However, the residues of HCHs and DDTs in almost all soil samples (except for surface soils in D30) in the three wetlands were below the guideline (GB 15618-1995) of the first criteria (50 ng g^−1^) recommended by the Chinese government to protect the natural environment and ecology by maintaining natural background levels^51^.

## Conclusions

This study investigated the occurrence, potential sources and ecological risks of organochlorine pesticides (OCPs) in soil profiles from ditch wetlands, riverine wetlands and reclaimed wetlands along a 100-yr chronosequence of reclamation in the Pearl River Estuary in China. Overall, ∑HCHs, ∑DDTs, HCB, and ∑DRINs were the dominant OCP isomers in the three wetlands, and total OCP concentrations decreased along the soil profiles in each of the three wetlands. Higher OCP levels were observed in ditch wetlands compared to riverine and reclaimed wetlands, indicating ditch wetlands might be an important site for OCPs. Compared to the D100 and D40 wetlands, a short-term wetland reclamation history (i.e., D30 and D20) contributed to OCP accumulation in ditch wetlands, whereas the lowest OCP levels were observed in D10 soils. The older reclaimed wetland soils had higher OCP levels than the more recently reclaimed ones. Comparatively, higher OCPs were observed in the younger riverine wetland soils. The more recent usage of lindane was the dominant source of *γ*-HCH, and DDT input was historical, with the current application of dicofol as an important potential source. Aldrin and endrin might still be used in this region based on the higher percentage of aldrin in these soils and endrin in the surface soils of the D20 wetlands. Compared to DDTs, HCHs had higher ecotoxicological risks in this region and should cause more concern. Surface soils in the three wetlands exhibited higher ecological risks, with OCPs exceeding TEL levels in more than 60% of the soils, whereas deeper soils showed no or lower ecological risks. Compared to riverine and reclaimed wetlands, ditch wetlands had higher ecotoxicological risks for OCPs. Finally, investigation of the effects of wetland hydrology on the levels, distribution, and ecological risks of OCPs due to spatial and temporal changes in hydrological conditions is still needed.

## Materials and Methods

### Site description

The study area is located at Wanqingsha of the Pearl River Estuary of China (22°36′39″ to 22°44′36″ N and 113°23′42″ to 113° 38′34″ E). It has a sub-tropical marine climate. The annual mean temperature is 21.9 °C, and the annual mean rainfall is 1647.5 mm. This region has suffered from heavy reclamation for more than 100 years. It is estimated that approximately 5200 ha of wetlands have been reclaimed in this region to date and the dominant land-use types in this region include riparian wetlands (flowing from northwest to the south sea along the western edge of the reclaimed land), ditch wetlands (ditches run across the reclaimed land to irrigate and drain the soils through water gates) and reclaimed wetlands (used to plant bananas, vegetables etc.)^21^,^52^. These tidal flat wetlands have been periodically reclaimed during different periods; i.e., a century ago (100-yr reclamation history), during the 1970s (approximately 40-yr reclamation history), 1980s (approximately 30-yr reclamation history), 1990s (approximately 20-yr reclamation history) and 2000s (approximately 10-yr reclamation history)^21^,^25^,^52^.

We selected three sampling belts along a 100-yr chronosequence of reclamation based on land use types and reclamation histories in the Pearl River Estuary. Five sampling sites each were set in riparian wetlands (i.e., Ri100, Ri40, Ri30, Ri20, and Ri10), ditch wetlands (i.e., D100, D40, D30, D20, and D10) and reclaimed wetlands (i.e., Re100, Re40, Re30, Re20, and Re10) along a 100-yr chronosequence of reclamation, which have approximately 100−, 40−, 30−, 20− and 10-yr reclamation histories, respectively (Supplementary information Figure S1). Soils in the ditch wetlands are classified as Eutric Gleysol, soils in the riparian wetlands are classified as Mollic Fluvisol, and soils in the reclaimed wetlands are classified as cropland soils^53^.

### Soil sample collection

Three sampling sites were randomly selected in each three soil cores (4.8 cm in diameter), with depths of 30 cm randomly collected at each sampling site within a 20 m radius along a 100-yr chronosequence of reclamation. The cores were stratified into three layers at 10 cm intervals and mixed with the same layer from each wetland type. All soil samples were placed in polyethylene bags and brought to the laboratory. All of the soil samples were air dried for three weeks at room temperature and sieved through a 2 mm nylon sieve to remove coarse debris and stones. All of the air-dried subsamples were ground with a pestle and mortar until all of the particles passed through a 0.149 mm sieve.

### Chemical analyses

Accelerated solvent extraction method (ASE300, Dionex, America) was adopted to extract 16 OCPs including α-HCH, β-HCH, γ-HCH, *p,p’*-DDD, *o,p’*-DDT, *p,p’*-DDT, *p,p’*-DDE, HCB, heptachlor, heptachlor epoxide, α-endosulfan, β-endosulfan, dieldrin, aldrin, endrin and MXC in soil samples (Supplementary information Table S1). A 20.00-g subsample was extracted with 30 ml of n-hexane/acetone (1:1, v/v) at 100 °C and 1500 psi in triplicate. Then, the extracts were concentrated to approximately 1 ml by rotary evaporation with a flow of N_2_ stream for cleanup. The concentrated extract was cleaned using a chromatography column (30 cm × 10 mm i.d.) with 2 g of silver nitrate silica (10% concentrated silver nitrate, wt/wt), 1 g of activated silica gel, 3 g of basic silica gel, 1 g of activated silica gel, 4 g of acidic silica gel (22% concentrated sulfuric acid, wt/wt), 1 g of activated silica gel and 2 g of anhydrous sodium sulfate. Elution was performed with 100 ml of hexane. The collected eluent was concentrated to 1 ml by rotary evaporation and then reduced to 1 ml under a gentle purified N_2_ stream for analysis.

OCPs were analyzed on an Agilent 6890 gas chromatograph (Wilmington, DE, USA) equipped with a micro-electron-capture detector (micro-ECD). The separation was carried out using an HP-5 capillary column (30 m × 0.25 mm × 0.25 μm), and high purity helium (99.9999%) was used as the carrier gas in constant flow mode. A volume of 1 μl was injected in automatic splitless mode. The injector port and detector temperatures were maintained at 260 °C and 280 °C, respectively. The column temperature was initially maintained at 60 °C for 1 min, increased to 140 °C at a rate of 10 °C min^−1^ and held for 1 min. The temperature was then increased again to 230 °C at a rate of 1.0 °C min^−1^, increased to 280 °C at a rate of 10 °C min^−1^ and then held for 21 min. Concentrations of individual OCPs were obtained based on the internal standard peak area method and a 6-point calibration curves for the individual components^2^.

### Quality control and quality assurance

Concentrations are reported on a wet weight basis (wet wt.), and those below the method detection limits are indicated as <MDL (MDL = 0.02 ng g^−1^ dry wt. for OCPs). The method performance was assessed through rigorous daily internal quality control, including regular analysis of certified materials. The precision of the measurements obtained through replicates of the reference materials was better than 10% for all target compounds. All data were subject to strict quality assurance and control procedures. For each set of 8 samples, a procedural blank and certified reference soil (AE-00051, AccuStandard) were used to determine the accuracy. The spiked recovery rates of OCP isomers ranged from 63% to 106%.

### Statistical analysis

One-Way ANOVA analysis was conducted to identify the differences in OCP isomers in the same soil depth between different wetland types and the differences in OCP isomers between different soil depths in each of three wetland types.

## Additional Information

**How to cite this article**: Bai, J. *et al*. Organochlorine pesticides (OCPs) in wetland soils under different land uses along a 100-year chronosequence of reclamation in a Chinese estuary. *Sci. Rep*. **5**, 17624; doi: 10.1038/srep17624 (2015).

## Supplementary Material

Supplementary Information

## References

[b1] Jones K. C. & Voogt D. P. Persistent organic pollutants (POPs): state of the science. Environ Pollut 100, 209–221, doi: 10.1016/S0269-7491 (1999). 15093119

[b2] Sun J. H., Feng J. A., Liu Q. & Li Q. L. Distribution and sources of organochlorine pesticides (OCPs) in sediments from upper reach of Huaihe River, East China. J. Hazard. Mater. 184, 141–146 doi: 10.1016/j.jhazmat.2010.08.016 (2010). 20828933

[b3] El-Shahawi M. S., Hamza, A., Bashammakh A. S. & Al-Saggaf W. T. An overview on the accumulation, distribution, transformations, toxicity and analytical methods for the monitoring of persistent organic pollutants. Talanta. 80, 1587–1597, doi: 10.1016/j.talanta.2009.09.055 (2010). 20152382

[b4] Yang D. . Residues of organochlorine pesticides (OCPs) in agricultural soils of Zhangzhou City, China. Pedosphere 22, 178–189 doi: 10.1016/S1002-0160 (2012).

[b5] Wang G. . 2009. Factors influencing the spatial distribution of organochlorine pesticides in soils surrounding chemical industrial parks. J. Environ. Qual. 38, 180–187 doi: 10.2134/jeq2008.0004 (2009). 19141808

[b6] Mumtaz M. . Human health risk assessment, congener specific analysis and spatial distribution pattern of organochlorine pesticides (OCPs) through rice crop from selected districts of Punjab Province, Pakistan. Sci. Total. Environ. 511, 354–361 doi: 10.1016/j.scitotenv.2014.12.030 (2015). 25553549

[b7] Yang R. Q., Jiang G. B., Zhou Q. F., Yuan C. G. & Shi J. B. Occurrence and distribution of organochlorine pesticides (HCH and DDT) in sediments collected from East China Sea. Environ. Int. 31, 799–804 doi: 10.1016/j.envint.2005.05.027 (2005). 16024080

[b8] Crisp T. M. . Environmental endocrine disruption: an effects assessment and analysis. Environ. Health. Perspect 106, 11–56. doi: PMC1533291 (1998). 9539004 10.1289/ehp.98106s111 PMC1533291

[b9] Komprda J., Komprdova K., Sanka M., Mozny M. & Nizzetto L. Influence of climate and land use change on spatially resolved volatilization of persistent organic pollutants (POPs) from background soils. Environ. Sci. Technol. 47, 7052–7059 doi: 10.10.1021/es3048784 (2013). 23506564

[b10] Shahbazi A., Bahramifar N. & Smolders E. Elevated concentrations of pesticides and PCBs in soils at the Southern Caspian Sea (Iran) are related to land use. Soil. Sediment. Contam. 21, 160–175 doi: 10.1080/15320383.2012.649373 (2012).

[b11] Die Q. Q., Nie Z. Q., Huang Q. F. & Zhu X. H. Organochlorine pesticides (OCPs) in soils of Pearl River Delta, China. J. Agro-Environ. Sci. 33, 298–304 (2014).

[b12] Wang F. . Organochlorine pesticides in soils under different land usage in the Taihu Lake region, China. J. Environ. Sci. 19, 584–590 doi: 10.1016/S1001-0742 (2007). 17915688

[b13] Zhu X. H. . Distribution and characteristics of OCPs in surface soils in certain districts of Guangzhou. Res. Environ. Sci. 25, 519–525 (2012).

[b14] Hao H. T., Sun B. & Zhao Z. H. Effect of land use change from paddy to vegetable field on the residues of organochlorine pesticides in soils. Environ. Pollut. 156, 1046–1052 doi: 10.1016/j.envpol.2008.04.021 (2008). 18554761

[b15] Fu J. M. . Persistent organic pollutants in environment of the Pearl River Delta, China: an over view. Chemosphere. 52, 1411–1422 doi: 10.1016/S0045-6535 (2003). 12867171

[b16] Zhou R. B., Zhu L. Z., Yang K. & Chen Y. Y. Distribution of organochlorine pesticides in surface water and sediments from Qiantang River, East China. J. Hazard. Mater. 137, 68–75 doi : 10.1016/j.jhazmat.2006.02.005 (2006). 16540236

[b17] Liu M. . Organochlorine pesticides in surface sediments and suspended particulate matters from the Yangtze Estuary, China. Environ. Pollut. 156, 168–173 doi: 10.1016/j.envpol.2007.12.015 (2008). 18222021

[b18] Monirith I., Ueno D. & Takahashi S. Asia-Pacific mussel watch: Monitoring contamination of persistent organochlorine compounds in coastal waters of Asian countries. Mar. Pollut. Bull. 46, 281–300 doi: 10.1016/S0025-326X (2003). 12604061

[b19] Dou L. & Yang G. Y. Distribution characteristics and risk assessment of organochlorine pesticides in surface soils of Peal River Delta Economic Zone. Environ. Sci. 36, 2954–2963 (2015). 26592027

[b20] Zhou J. Y. . Sources, Transport and Environmental Impact of Contaminants in the Coastal and Estuarine Areas of China. China Ocean Press, Beijing (1997).

[b21] Xiao R. . Fractionation, transfer, and ecological risks of heavy metals in riparian and ditch wetlands across a 100-year chronosequence of reclamation in an estuary of China. Sci. Total Environ. 517, 66–75doi: 10.1016/j.scitotenv.2015.02.052 (2015). 25723958

[b22] Zhang G. . Sedimentary records of DDT and HCH in the Pearl River Delta, South China. Environ. Sci. Technol. 36, 3671–3677 doi: 10.1021/es0102888 (2002). 12322736

[b23] Allsopp M. & Johnston P. Unseen poisons in Asia: A review of persistent organic pollutant levels in South and Southeast Aisa and Oceanic. 2000—03, http://www.greenpeace.org/international/Global/international/planet-2/report/2000/2/unseen-poisins-in-asia-a-rev.pdf. (2000) (Date of access: 20/10/2015).

[b24] Iwata H., Tanabe S., Sakai N., Nishimura A. & Tatsukawa R. Geographical distribution of persistent organochlorines in air, water and sediments from Asia and Oceania, and their implications for global redistribution from lower latitudes. Environ. Pollut. 85, 15–33 doi: 10.1016/0269-7491 (1994). 15091681

[b25] Bai J. H. . Soil organic carbon as affected by land use in young and old reclaimed regions of a coastal estuary wetland, China. Soil Use Manage 29, 57–64 doi: 10.1111/sum.12021 (2013).

[b26] Wu Q. . Biological risk, source and pollution history of organochlorine pesticides (OCPs) in the sediment in Nansha Mangrove, South China. Mar. Pollut. Bull. 96, 57–64 doi: 10.1016/j.marpolbul.2015.05.047 (2015). 26021291

[b27] Da C. N. . Sources and Risk Assessment of Organochlorine Pesticides in Surface Soils from the Nature Reserve of the Yellow River Delta, China. Soil. Sci. Soc. Am. J. 78, 779–786 doi: 10.1007/ s11356-013-2269-6 (2014).

[b28] Hu W. Y., Huang B., Zhao Y. C., Sun W. X. & Gu Z. Q. Organochlorine pesticides in soils from a typical alluvial plain of the Yangtze River Delta region, China. Bull. Environ. Contam. Tocxicol. 87, 561–566, doi: 10.1007/s00128-011-0368-y (2011). 21785876

[b29] Wang L. . Historical contamination and ecological risk of organochlorine pesticides in sediment core in northeastern Chinese river. Ecotoxicol. Environ. Saf. 93, 112–120 doi: 10.1016/j.ecoenv.2013.04.009 (2013) 23683900

[b30] Lin T. . Levels and mass burden of DDTs in sediments from fishing harbors: the importance of DDT-containing antifouling paint to the coastal environment of China. Environ. Sci. Technol. 43, 8033–8038 doi: 10.1021/es901827b (2009). 19924919

[b31] Barber J., Sweetman A. & Jones K. Hexachlorobenzene-sources, environmental fate and risk characterization. Science Dossier. Euro Chlor. (2005).

[b32] Nyagode B. A., Jame M. O. & Kleinow K. M. Influence of dietary co-exposure to Benzo(a)pyrene on the biotransformation and distribution of 14C-Methoxychlor in the Channel catfish (Ictalurus punctatus). Toxicol. Sci. 108, 320–329 doi: 10.1093/toxsci/kfp018 (2009). 19181613 PMC2664691

[b33] Qu C. K. . Risk assessment and influence factors of organochlorine pesticides (OCPs) in agricultural soils of the hill region: A case study from Ningde, southeast China. J. Geochem. Explor 149, 43–51 doi: 10.1016/j.gexplo.2014.11.002.(2015).

[b34] Gao J., Zhou H., Pan G., Wang J. & Chen B. Factors influencing the persistence of organochlorine pesticides in surface soil from the region around the Hongze Lake, China. Sci. Total Environ. 443, 7–13 doi: 10.1016/j.scitotenv.2012.10.086 (2013). 23178885

[b35] Lee K. T., Tanabe S. & Koh C. H. Distribution of organochlorine pesticides in sediments from Kyeonggi Bay and nearby areas, Korea. Environ. Pollut 114, 207–213 doi: 10.1016/S0269-7491 (2001). 11504343

[b36] Walker K., Vallero D. A. & Lewis R. G. Factors influencing the distribution of lindane and other hexachlorocyclohexanes in the environment. Environ. Sci. Technol. 33, 4373–4378 doi: 10.1021/es990647n (1999).

[b37] Li Y. F., Cai D. J. & Singh A. Technical hexachlorocyclohexane use trends in China and their impact on the environment. Arch. Environ. Contam. Toxicol. 35, 688–697 doi: 10.1007/s002449900432 (1998). 9776788

[b38] Alonso-Hernandez C. M., Mesa-Albernas M. & Tolosa I. Organochlorine pesticides (OCPs) and polychlorinated biphenyls (PCBs) in sediments from the Gulf of Batabanó, Cuba. Chemosphere 94, 36–41 doi: 10.1007/s002449900267 (2014). 24103440

[b39] Hites R. K. & Day H. R. Unusual persistence of DDT in some western USA soils. Bulletin Environ. Contam. Toxic. 48, 259–264. doi: 10.1007/BF00194381 (1992). 1536998

[b40] Hong H. S., Chen W. Q., Xu L., Wang X. H. & Zhang L. P. Distribution and fate of organochlorine pollutants in the Pearl River Estuary. Mar. Pollut. Bull. 39, 376–382 doi: 10.1016/S0025-326X (1999).

[b41] Zhou J. L., Maskaoui K., Qiu Y. W., Hong H. S. & Wang Z. D. Polychlorinated biphenyl congeners and organochlorine insecticides in the water column and sediments of Daya Bay, China. Environ. Pollut. 113, 373–384 doi: 10.1016/S0269-7491 (2001). 11428145

[b42] Tao S., Li B. G., He X. C., Liu W. X. & Shi Z. Spatial and temporal variations and possible sources of dichlorodiphenyltrichloroethane (DDT) and its metabolites in rivers in Tianjin, China. Chemosphere 68, 10–16, doi: 10.1016/j.chemosphere.2006.12.082 (2007). 17292453

[b43] Qiu X. H., Zhu T., Yao B., Hu J. X. & Hu S. W. Contribution of dicofol to the current DDT pollution in China. Environ. Sci. Technol 39, 4385–4390. doi: 10.1021/es050342a (2005). 16047771

[b44] Thomas J. E., Ou L. T. & Al-Agely A. DDE remediation and degradation. Rev. Environ. Contam. Toxicol. 194, 55–69 doi: 10.1007/978-0-387-74816-0_3 (2008). 18069646

[b45] Agency for Toxic Substances and Disease Registry (ATSDR). Toxicological profile for aldrin and dieldrin. U.S. Public Health Service. Atlanta, G.A. (2002). 37040456

[b46] Said T. O., Okbah M. A., Mohamed L. A. & Othman I. M. Detection of persistent OCPs and PCBs congeners in the near-shore coastal waters of Alexandria, Egypt. Environ. Monit. Assess. 187, 353 doi: 10.1007/s10661-015-4537-z (2015). 25971520

[b47] Long E. R., MacDonald D. D., Smith S. L. & Calder F. D. Incidence of adverse biological effects within ranges of chemical concentrations in marine and estuarine sediments. Environ. Manage 19, 81–97 doi: 10.1007/BF02472006 (1995).

[b48] Jongloed R. H., Traas T. P. & Luttik R. A probabilistic model for deriving soil quality criteria based on secondary poisoning of top predators: II Calculations for dichlorodiphenyltrichloroethane (DDT) and cadmium. Ecotox. Environ. Safe 34, 279–306, doi: 10.1006/eesa.1996.0072 (1996). 8812196

[b49] Urzelai A., Vega M. & Angulo E. Deriving ecological risk-based soil quality values in the Basque County. Sci. Total. Environ. 247, 279–284 doi: 10.1016/S0048-9697(2000). 10803555

[b50] Netherlands Ministry of Housing. Spatial planning and environment’s circular on target values and intervention values for soil remediation. http://www.esdat.net/Environmental%20Standards/Dutch/annexS_I2000Dutch%20Environmental%20Standards.pdf (2002) (Date of access: 20/10/2015).

[b51] Science & Technology Department of State Environmental Protection. Environmental quality standard for soils. http://kjs.mep.gov.cn/hjbhbz/bzwb/trhj/trhjzlbz/199603/W02007031348558 7994018.pdf (1995) (Date of access: 20/10/2015).

[b52] Cui Z. H. Effect of reclamation on chemical species of heavy metals in tidal flat soil from the Pearl River Estuary. Master thesis, Jinan University (2010).

[b53] IUSS, ISRIC & FAO. World Soil Resources Reports: World Reference Base for Soil 625Resources, A Framework for International Classification, Correlation and ommunication. 626 FAO, Rome (2006).

