# Supplementary material for: Organochlorine pesticides (OCPs) in wetland soils under different land uses along a 100-year chronosequence of reclamation in a Chinese estuary
Source: Sci Rep. 2015 Dec 3;5:17624. doi: 10.1038/srep17624 (PMC4668565; doi:10.1038/srep17624)
Supplement: Supplementary Information [file srep17624-s1.doc]

**Organochlorine pesticides (OCPs) in wetland soils under different land uses along a 100-year chronosequence of reclamation in a Chinese estuary**

Junhong Bai[[1]](#footnote-2)*, Qiongqiong Lu, Qingqing Zhao, Junjing Wang, Zhaoqin Gao, Guangliang Zhang

State Key Laboratory of Water Environment Simulation, School of Environment, Beijing Normal University, Beijing 100875, P.R. China

Table S1 Summary of detected OCP isomers in the top 10cm soils of three wetland types

| OCP isomers | Ditch wetlands | Riverine wetlands | Reclaimed wetlands |
| --- | --- | --- | --- |
| α-HCH | Nd-4.77(1.37) | Nd-3.33(2.23) | Nd-2.34(1.59) |
| β-HCH | Nd-4.59(1.75) | 1.40-2.47(2.03) | 1.59-2.53(2.11) |
| γ-HCH | Nd-3.58(1.40) | 2.78-8.01(5.56) | 2.90-4.10(3.40) |
| p,p'-DDD | 1.48-2.77(2.06) | 1.34-2.39(1.86) | Nd-2.09(1.43) |
| o,p'-DDT | Nd-6.94(3.17) | 1.71-9.47(3.77) | Nd-1.80(0.68) |
| p,p'-DDT | Nd-396.85(87.15) | Nd-6.93(1.39) | Nd-8.31(2.54) |
| p,p'-DDE | 1.57-8.24(4.10) | 2.34-4.50(3.33) | Nd-3.76(2.37) |
| HCB | 4.15-332.56(93.69) | 13.03-26.32(19.31) | 4.02-55.40(18.15) |
| Heptachlor | Nd-3.91(1.48) | Nd | Nd |
| Heptachlor epoxide | Nd-2.18(0.95) | Nd-1.37(0.27) | Nd-2.26(1.35) |
| α-ENdosulfan | Nd-1.22(0.47) | Nd | Nd-1.31(0.26) |
| β-ENdosulfan | Nd-1.9(0.38) | Nd-1.99(0.78) | Nd-2.18(1.23) |
| Dieldrin | Nd-2.19(1.53) | 1.50-3.17(2.38) | Nd-2.42(1.31) |
| Aldrin | Nd-29.11(14.25) | 10.31-34.58(21.03) | 3.56-10.68(7.17) |
| ENdrin | Nd-8.35(3.78) | 2.42-15.83(5.46) | Nd-3.70(2.07) |
| MXC | Nd-116.15(48.65) | 3.85-7.54(4.74) | Nd-3.46(2.03) |
| ∑OPCs | 15.87-635.16(266.18) | 54.21-94.32(73.67) | 12.45-99.53(47.69) |
|  |  |  |  |

Nd : not detected. Value in the bracket is the mean.


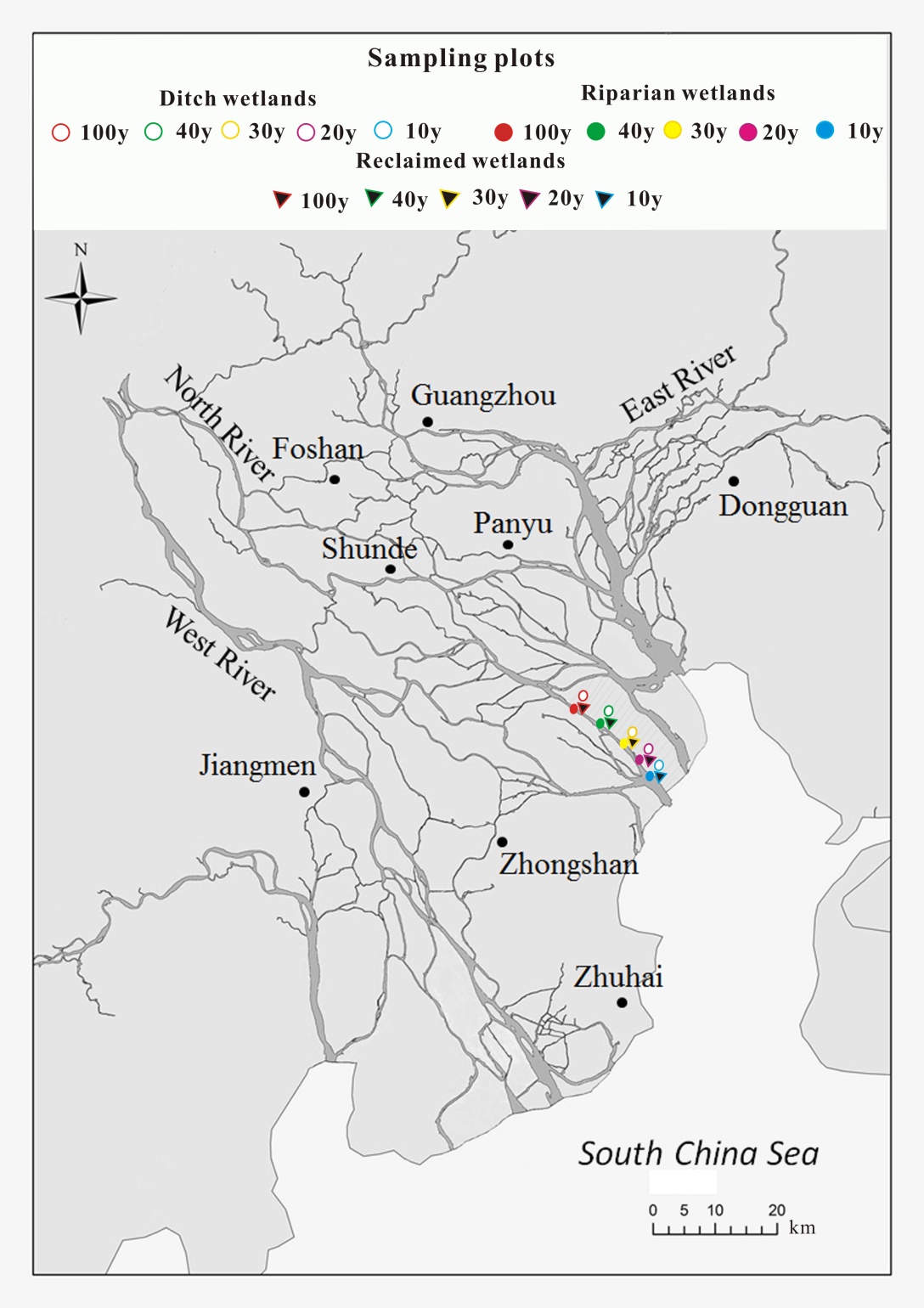


Figure S1 Location map of sampling plots in the study area. This map is extracted from Landsat TM image of the Pearl River Estuary using EVI4.8 software package and created using ArcGIS 10.2 and Coreldraw X4 software packages by the second author (Q.L.).

1. ^^ Corresponding author. Dr. Junhong Bai, [Tel: +86-010-58802029](tel:86-010-58802029), Fax:+86-010-58800397, Email: [baijh@126.com](mailto:baijh@126.com) [↑](#footnote-ref-2)
